# From inclusion to empowerment: advancing equity through co-research with people living with dementia

**DOI:** 10.3389/frdem.2025.1600162

**Published:** 2025-05-13

**Authors:** Lillian Hung, Joey Wong, Karen Lok Yi Wong, Emily Ong, Granville Johnson, Helen Rochford-Brennan, Jim Mann, Lester Gierach, Lynn Jackson, Mario Gregorio, Mary Beth Wighton, Phyllis Fehr

**Affiliations:** ^1^School of Nursing, University of British Columbia, Vancouver, BC, Canada; ^2^School of Social Work, University of British Columbia, Vancouver, BC, Canada; ^3^IDEA Lab, School of Nursing, University of British Columbia, Vancouver, BC, Canada; ^4^Behavioural Supports Ontario, North Bay, ON, Canada

**Keywords:** empowerment, Patient and Public Involvement (PPI), dementia research, patient engagement, health research

## Abstract

Too often, people living with dementia are spoken for rather than spoken with. This reflects deeply embedded assumptions/biases about people living with dementia in our society. This article explores the experiences and insights of individuals with dementia, positioning empowerment as a foundational strategy to advance social citizenship and equity. We collaboratively on more than a decade of shared work in research and advocacy. Our collective analysis identifies three key dimensions essential for meaningful empowerment: (a) recognizing strengths and building capacities, (b) equitable leadership, and (c) supportive environments and learning together. Our findings demonstrate that intentional, respectful collaboration produces extensive social, political, and healthcare benefits, actively challenging inequities and fostering a deeper sense of belonging and contribution.

## Introduction

Social citizenship demands that individuals with lived experience are recognized not only as care recipients or research subjects but as full participants in society, with the right to shape the decisions, policies, services, and research that directly affect their lives. Empowerment is a key condition for the realization of this form of citizenship (Reis et al., 2022). Yet, despite its widespread use in both practice and scholarship, the concept of empowerment remains inconsistently defined, particularly in relation to people living with dementia. Historically, dominant assumptions about cognitive decline and incapacity have led to the exclusion of people living with dementia from defining the very terms and conditions of their empowerment.

This trend is shifting. In recent years, co-researchers and activists living with dementia have challenged the marginalization of their voices, calling attention to the structural barriers that limit their agency and participation. McConnell et al. (2019) for example, co-developed a definition of empowerment directly with people living with dementia. They describe it as “a confidence-building process whereby people living with dementia are respected, have a voice and are heard, are involved in making decisions about their lives, and have the opportunity to create change through access to appropriate resources (p. 1).” Similarly, van Corven et al. (2021) synthesized the literature to highlight key elements of empowerment, including choice, control, autonomy, the use of one’s abilities, active participation, self-worth, confidence, and meaningful social relationships. Importantly, they underscore that empowerment does not exist in a vacuum — it is shaped by environmental conditions such as stigma, public attitudes, and access to supportive relationships.

These insights resonate strongly with the literature on Patient and Public Involvement (PPI), which emphasizes the value of engaging people with lived experience as partners in research. As Schilling and Gerhardus (2024) note, empowerment is an outcome of meaningful involvement and a guiding principle — emphasizing participation, challenging traditional power structures, recognizing diverse forms of knowledge, and fostering collaborative relationships. The ethical imperative is clear: people must be involved in decisions that affect their lives. As Charlton (1998) so powerfully states, “Nothing about us without us.”

Beyond ethics, meaningful engagement improves research quality. Lived experience provides unique insights that will enhance findings’ relevance, credibility, and practical applicability (Boote et al., 2010; Brett et al., 2014; Gradinger et al., 2015). However, achieving genuine empowerment in research is far from straightforward. Scholars have noted persistent challenges — tokenism, lack of structural change, and the superficial involvement of people with dementia in research processes (Schilling and Gerhardus, 2024; Halvorsen et al., 2020). Tokenism in co-research refers to the superficial inclusion of individuals—such as people with lived experience—without granting them meaningful influence or decision-making power in the research process (Mann and Hung, 2019). When involvement is symbolic rather than substantive, it can reinforce feelings of exclusion and disempowerment, ultimately discouraging future engagement.

This paper addresses the question, “How can we meaningfully conceptualize and enact empowerment in the context of research with people living with dementia?” Drawing from more than a decade of co-research with individuals living with dementia, we examine how empowerment can be fostered through collaborative inquiry. We reflect on the barriers that undermine this goal while identifying practical strategies to support empowerment in research relationships. Grounded in the lived experience of dementia, our work aims to advance a more equitable, inclusive, and socially just research practice.

We are a team with deep and diverse experience in dementia advocacy, research, education, and clinical practice co-authors this paper. The research team brings together individuals of varied gender identities and ethnocultural backgrounds, reflecting a commitment to inclusivity and intersectional perspectives. Intersectionality recognizes that people’s experiences are shaped by multiple, overlapping social identities and systems of oppression, such as racism, sexism, ageism, and ableism; this lens helps ensure that research reflects the complexity of lived experiences and addresses structural inequities (Crenshaw, 1989; McCall, 2005; Giebel, 2024). Central to this collaboration is ten people living with different types and stages of dementia, whose lived expertise grounds the work in real-world insight and relevance. The co-authors living with dementia are deeply engaged in efforts to improve the quality of life for people with dementia globally. They are active contributors to national and international research initiatives focused on stigma reduction, cultural and racial inclusivity, dementia-friendly environments, and emerging technologies in care. Their work spans public speaking, policy advising, and mentorship of students and early-career researchers. They also co-author academic papers, lead community outreach efforts, and serve on advisory committees that influence service design and social policy. This collaborative authorship exemplifies the value of meaningful inclusion — not merely inviting people living with dementia to share experiences but co-producing knowledge with them as equal partners. Their leadership in shaping this paper affirms the potential of empowerment through research.

## The collaborative reflection process

The initial reflection questions were developed by LH based on the concepts around social citizenship and empowerment in the literatures. All questions were then reviewed and refined collaboratively by LH and MG, a co-author living with dementia, into more accessible languages to be used in the reflection sessions. Some examples of the questions are listed in Table 1. As all co-authors are situated in different places around the globe, we had separate discussions and dialogues to accommodate of our time zones. We had seven group reflection sessions, with four to seven co-authors in each session. The sessions were conducted on Zoom and in-person meetings. The discussions were co-facilitated by LH, MG and JW. The co-authors validated, expanded and discussed diverse aspects of empowerment during the reflection sessions. Eventually, they were finalized into three interrelated aspects which will be elaborated below.

**Table 1:** Examples of the reflection questions.

1. How do you feel about taking part in research or helping others with advocacy work?
2. What needs to change so people with dementia feel more included and appreciated in society?
3. How can researchers better see and support what you are good at?
4. What makes you want to get involved in research or advocacy work? What makes it hard to join in?
5. What kinds of opportunities or help do you think are important to make people with dementia feel part of things?

### What are the key aspects of empowerment?

From these reflections, we identified three interrelated aspects of empowerment: (a) recognizing strengths and building capacities, (b) equitable leadership, (c) supportive environments and learning together.

#### Recognizing strengths and building capacities

A strengths-based approach begins by affirming the abilities, knowledge, and lived experiences of people with dementia, positioning them not as passive recipients of care or knowledge but as contributors, leaders, and change-makers. Rather than focusing on deficits, this approach asks: What can each person offer? How can we meaningfully recognize and support their strengths? Central to this ethos is the understanding that expertise comes in many forms and that lived experience is a powerful form of knowledge in research and advocacy.

Recognizing strengths involves slowing down, listening deeply, and finding creative ways to meet people where they are, attuning to their interests, communication styles, and goals. It also involves shifting the research culture from one of evaluation to one of collaboration, where people living with dementia are welcomed as equal partners. As Phyllis reflected, her work contributing to the Canadian dementia strategy, including participation in advocacy at the United Nations, has led to tangible change, “It has led to government funding investment and so many good things,” she explained. “But it’s still missing a lot of things, such as changes happening at the individual level, helping the people living in their own homes and struggling.” Her comment underscores the dual focus of empowerment: structural advocacy is essential, but so too is attending to the everyday realities of people living with dementia.

Confidence-building also demands an environment of psychological safety. Mistakes must be normalized as part of learning, not as sources of embarrassment. Mario shared a moment from an international conference where he raised his hand to ask a question, only to forget it partway through: “I forgot to write it down because it was so fast, and you have only one opportunity to raise your hand and be selected. So, I raised my hand, and after one sentence, I was about to ask the question, but I forgot the question. Everybody laughed. I know everybody was with me. It was not an embarrassing moment. I do not let embarrassment stop me from joining these conversations.” His story exemplifies how communal support and respectful environments enable individuals to engage with courage and enthusiasm, even when challenges arise.

Creativity is vital in ensuring that strengths are not lost to structural barriers. For instance, voice-to-text tools, speech preparation apps, and iPad-based research materials enabled Phyllis to actively participate in writing, organizing, and presenting complex information. These adaptations were not viewed as compensations for loss but as enablers of participation. By providing the right support, people living with dementia can engage in all aspects of research, from study design and data collection to dissemination and impact planning.

This reorientation toward capacity, rather than incapacity, directly challenges the deficit-based narratives that continue to stigmatize dementia. It also affirms that people living with dementia can and do lead, teach, and shape knowledge. Embracing a strengths-based approach moves us closer to equity by redefining what contribution looks like and fostering conditions where people can thrive.

#### Equitable leadership

Democratic leadership calls for reimagining traditional research hierarchies shifting toward shared power and decision-making models. Within this framework, the voices of people living with dementia are not supplementary; they are central. Equitable leadership means co-creating the agenda, sharing responsibility, and fostering an environment where everyone’s knowledge and contributions are valued equally. Too often, people living with dementia are present in name but absent in influence. Democratic leadership addresses this imbalance by fostering collaborative spaces where power dynamics are visible and consciously renegotiated. It is a process rooted in co-leadership, mutual accountability, and trust. When people living with dementia are recognized as leaders, mentors, and decision-makers, the result is not only a more ethical process; it also produces more meaningful and impactful outcomes.

For Colin, being an active contributor rather than a passive observer brought a profound sense of purpose and achievement. His experiences in intergenerational research projects attending in-person meetings and community events, demonstrated how inclusive environments cultivate energy, motivation, and continued engagement. He shared that being included inspired him to take on further advocacy work, showing how active involvement invites sustained commitment. Similarly, members of our team living with dementia expressed a desire to go beyond participation. They wanted to mentor trainees, co-design research, and contribute to broader community impact. These aspirations speak to a key tenet of democratic leadership: avoiding tokenism. People do not want to be included symbolically; they want their insights to shape the direction and consequences of the work.

At the same time, the absence of genuine inclusion can cause harm. Granville reflected on experiences of being sidelined or disregarded in collaborative settings, describing how exclusion can lead to frustration, burnout, and a breakdown of trust. “As a researcher, you must truly be willing to practice active listening and adopt an open-minded approach,” he emphasized. “There are very few Black people living with dementia who feel comfortable speaking openly about their condition. It’s a trust issue because it puts you in an extremely vulnerable position. Transparency and respecting the direct words of people with dementia are essential. Disregarding their contributions undermines trust and the value they bring to the team.” His reflection highlights how the stakes of exclusion are especially high for racialized individuals living with dementia, who may already face compounded barriers to participation due to stigma, systemic bias, or previous harmful experiences.

Building long-term, meaningful relationships is essential. Granville noted that ongoing trust requires small acts of care and relational maintenance, such as checking in or sending a holiday card. These gestures communicate respect and help ensure engagement is not extractive or opportunistic. As he warned, “People hop on, have a little ride, then they hop off.” This metaphor captures how people living with dementia are too often treated as temporary consultants rather than enduring partners. Democratic leadership resists this trend by embedding people with lived experience into the fabric of research and advocacy teams. By fostering trust, transparency, and shared ownership, democratic leadership transforms who gets to lead and how leadership itself is defined. Lynn offered a nuanced view of shared governance, “There needs to be that conversation about what you can bring to the table. But also, you need to be able to tell the researcher what you cannot bring to the table... quite often, we know—but there are some things we do not know yet.”

#### Supportive environments and learning together

Creating supportive environments is essential for enabling people living with dementia to participate meaningfully in research and advocacy. The environments mentioned in this theme refer to physical or social spaces when people living with dementia come together to do things meaningful and significant to them, such as research, networking opportunities and advocacy. Such environments must be intentionally designed to provide opportunities, resources, and relational support that empower individuals to engage with confidence, creativity, and purpose. As Emily pointed out, stigma continues to create invisible but powerful barriers, “Most of the time, there is no opportunity given to people with dementia. After the diagnosis, there is a stigma that people are no longer competent. When there is no opportunity given, it is unlikely the person with dementia can discover they have the potential to contribute.”

Equitable opportunity to contribute, including technology, transportation, and knowledge—enables people living with dementia to contribute fully and confidently. For example, in the social robot project involving LOVOT social robots, Mario described the joy participants experienced through simple, tangible interactions that brought people together. Similarly, Emily shared how she helped a peer overcome internalized stigma by inviting him to attend public events, where repeated practice helped him build confidence. Mario added that being open to possibilities and inviting people into roles like tour guides—even if they make mistakes—can be a powerful way to build confidence and community.

These opportunities are not only about engagement; they are about connection. Helen shared, “The feeling and experience of caring for each other is the impact of empowerment.” This emphasis on care and relationships reflects a broader understanding of empowerment as inherently social. For Lester, participating in a ukulele project highlighted how even playful, low-stakes environments can be significant, “I enjoyed it because it was fun to be part of the conversations with others. You could even make mistakes, and it would be OK.” These experiences foster inclusion and belonging, which are central to individual well-being and collective impact.

Sustaining empowerment over time requires networks of support. Phyllis spoke about the value of intergenerational allies, while Jim emphasized proactive participation, “Not waiting for an invitation but bringing yourself to the table.” In the face of stigma, empowerment helps people reclaim a sense of personhood, reminding them—and the world—that they are more than a diagnosis.

Learning together is another crucial element of supportive environments. Empowerment is not a one-way transaction but a reciprocal process through which researchers and people living with dementia teach and learn from one another. MaryBeth emphasized the importance of involving people with dementia early in the research process, while Jim acknowledged that researchers may not always realize the value of lived experience until later. His story illustrated how people living with dementia can create pivotal learning moments for researchers, helping them recognize the depth and significance of their perspectives. Helen explained the impact of this mutual learning on identity, “It gives me a positive identity beyond the diagnosis. I’m more than a diagnosis.” Jim added, “It’s about taking ownership, grabbing the torch, and actively moving ahead as an active citizen.” MaryBeth deepened this metaphor, “We went out and found our torch to ignite us and build a legacy, to make that torch as bright as we can as individuals. It can be a very dark world without hope.” These reflections underscore that learning is not just about acquiring knowledge—it is about reclaiming agency, shaping identity, and building hope.

Emily described her relational approach to education and advocacy, “I always give that power and ownership to the learners because they are the ones who discover the learning. My job is to facilitate, and we learn together... ‘Nothing about me without me’ is very important. If you want to talk about dementia, please engage someone who is living with dementia... but also remember: nothing about them without them.” Her words emphasize that collaboration is not simply about inclusion—it is about mutual respect, shared responsibility, and a deep commitment to co-creation. Fostering supportive environments means being curious about others, recognizing that each individual—researcher or advocate—brings unique ways of learning and contributing. As Emily reminded us, asking questions like “Where do their ideas come from?” is a way of honouring differences and encouraging genuine understanding. Regular mutual feedback, collaborative reflection, and adaptability to differences are essential to ensure that partnerships evolve in empowering, respectful, and transformative ways for all involved.

## Discussion

Empowerment is a relational and dynamic process that takes root within networks of trust, mutual respect, and shared learning. Supportive environments are essential for this process to unfold. When individuals feel valued, heard, and included, they can participate meaningfully, contribute confidently, and grow both individually and collectively. Empowerment is neither symbolic nor abstract in this context—it is tangible, practical, and deeply personal.

A central implication of this paper is that empowerment must be built on respect for lived experience. Participation must be meaningful and impactful. When people living with dementia are engaged in research and advocacy, they can amplify their voices, challenge dominant narratives, and reshape societal understandings of dementia. Building confidence and recognizing individual strengths emerged as vital components of empowerment. These are not secondary benefits but central mechanisms through which people with dementia develop a positive sense of identity. When supported in leadership roles, co-authorship, and public speaking, individuals become agents of change—both for themselves and their communities. As Lynn emphasized, empowerment requires creating inclusive spaces where all contributions are seen as important and where diverse perspectives are welcomed and respected. Her insights remind us that relationality is the foundation of empowerment. The relationality perspective aligns with the emphasis on facilitation reported by members of the Dementia Empowerment Group (McConnell et al., 2019).

MaryBeth expanded this idea by positioning empowerment as a vehicle for structural transformation. For her, empowerment includes the ability to influence policies, shape research agendas, and demand environments responsive to the needs of people with dementia. This level of influence requires not only individual confidence but also systemic change. Researchers and institutions must be willing to examine their practices—including confronting personal biases related to race, gender, and sexual orientation—to ensure that their work is inclusive, equitable, and just. People from marginalized communities face additional barriers. For racialized individuals and LGBTQ+ people living with dementia, stigma, exclusion, and systemic bias further complicate their participation in research and care. Constant individual and group critical reflections are essential by everyone in the team to avoid reproducing these inequities. Empowerment may be a buzzword, but it is an important concept about enabling people. It means to embrace the abilities we have, and it gives a sense of hope to others by being able to speak out.

This imperative is particularly urgent given the increasing visibility of hate crimes and discrimination targeting LGBTQ+ individuals and other marginalized groups. Implicit and explicit biases in research and healthcare continue to perpetuate harm. Empowerment requires a proactive and steadfast commitment to equity at every stage of research and care—starting with whose voices are heard, whose knowledge is validated, and whose lives are prioritized.

As the United Nations Convention on the Rights of Persons with Disabilities reminds us, states are obligated to “promote, protect and ensure the full and equal enjoyment of all human rights and fundamental freedoms by all persons with disabilities, and to promote respect for their inherent dignity (United Nations, 2025).” Dementia is often referred to as a “hidden disability,” but it is time for that to change. Dignity must be visible. Voice must be recognized. Participation must be real.

To achieve this vision, empowerment cannot be left to chance or good intentions. It must be cultivated through intentional practices, shared decision-making, resource equity, and long-term relationships. The findings of this paper provide a framework for how this can be done, grounded in the lived experience of people living with dementia. What emerges is not just a model of empowerment—but a roadmap for building a more inclusive, just, and equitable society.

Drawing on the lived experiences and collective reflections of our co-researchers, we offer the acronym EMPOWER as a framework to guide equitable engagement and systemic change. EMPOWER stands for “Educate and Engage,” “Make Voices Heard,” “Provide Resources and Access,” “Open, Safe and Welcoming Spaces,” “Work with Intersectionality,” “Elevate Strengths” and “Reflect Critically and Challenge Assumptions.” Refer to Table 2 for further elaborations.

**Table 2:** Practical strategies for empowerment.

	Vision statements	Practical strategies
E – Educate and Engage	Empowerment begins with public education and intentional engagement. Many people living with dementia face the dual burden of stigma and invisibility—experiencing a hidden disability that is often misunderstood or dismissed.	Education must go beyond dispelling myths. It must actively highlight the capacities, contributions, and potential of people living with dementia. Mentorship, awareness campaigns, and training opportunities can equip individuals with dementia and their allies to challenge stereotypes, raise awareness, and build confidence. Advocacy training, in particular enables people to speak for themselves and with others, shaping conversations around dementia with authority and credibility.
M – Make Voices Heard	True empowerment demands that the voices of people living with dementia are not only welcomed but *sought out* and *prioritized*. This includes voices from marginalized communities who are often excluded from dominant narratives.	Inclusive decision-making processes must be established to ensure that lived experience informs research, policy, and service design. Participation should not be symbolic—it should shape outcomes. Creating intentional platforms for people with dementia to share their stories, insights, and recommendations allows their perspectives to lead, not follow, the change process.
P – Provide Resources and Access	Resources are a precondition for empowerment. When these supports are in place, people can participate more fully and meaningfully, enhancing their dignity, agency, and quality of life.	People living with dementia need access to the tools, technologies, and accommodations necessary to engage fully and comfortably. This includes assistive technologies, accessible communication formats, and flexible research timelines. It also includes support for care partners, who often play an essential role in enabling sustained engagement.
O – Open, Safe and Welcoming Spaces	Empowerment flourishes in environments where people feel safe, respected, and seen. Safety is both emotional and cultural; people must know that their contributions will be valued and that their identities will be affirmed.	Creating welcoming spaces means rejecting tokenism and judgment while cultivating trust, openness, and belonging. Regular check-ins, peer support, and inclusive facilitation strategies help build these environments. Responsibility for safety and inclusion is collective; it must be embraced by all members of a research or advocacy team.
W – Work with Intersectionality	Empowerment strategies must account for the layered and intersecting identities of people living with dementia. Race, gender, sexuality, socioeconomic status, disability, and migration history all shape how individuals experience dementia—and how they are treated by systems. As Granville expressed, “Experience as a Black man comes before experience of dementia.” This reminder underscores the importance of addressing multiple forms of oppression and exclusion. As one co-researcher stated in our conversations, “We cannot empower if we do not recognize diversity.” Empowerment must be intersectional to be equitable.	Empowerment requires researchers and institutions to move beyond one-size-fits-all approaches and respond to the complexity of real lives. It reminds everyone in the team to avoid assumptions.
E – Elevate Strengths	Empowerment is not only about correcting injustice but also about celebrating strength.	This includes recognizing the talents, skills, and knowledge of people living with dementia as assets, not anomalies. Supporting people to lead projects, mentor others, or contribute creatively can challenge stigmas and reshape public narratives. Highlighting stories of leadership, resilience, and impact reinforces a more inclusive understanding of dementia and fosters community confidence in people’s continued contributions as full citizens.
R – Reflect Critically and Challenge Assumptions	Empowerment requires ongoing critical reflection—both personal and structural. People living with dementia are not a monolith. Their needs, interests, and capacities evolve over time and across contexts.	Researchers and institutions must examine their assumptions about dementia, disability, and capacity, continually asking: *Whose voices are included? Whose are missing? What barriers remain unaddressed?* Regular reflection and evaluation, including the use of equity-focused indicators, can ensure that empowerment efforts remain responsive, inclusive, and impactful. Spaces for shared reflection also strengthen partnerships and support learning for all involved.

## Conclusion

Empowerment, a key aspect of social citizenship, in dementia research and advocacy is an evolving, relational process rooted in respect, inclusion, and trust. At its core, empowerment means recognizing people living with dementia as experts in their own right—capable of leading, shaping, and challenging the systems that affect their lives. This requires authentic engagement, where strengths are celebrated, relationships are nurtured, and direct voices are not just included but amplified. To move beyond symbolic participation, intentional strategies are needed to dismantle structural barriers and promote social inclusion. The EMPOWER framework outlined in this paper offers practical guidance for building environments that honour diversity, challenge stigma, and facilitate meaningful and sustained participation. Crucially, empowerment is not about doing something *for* people living with dementia; it is about co-creating possibilities *with* them. It involves recognizing the complexity of people’s identities and experiences while addressing systemic inequities that have long silenced or sidelined their contributions. To build a just and inclusive future, we must move beyond inclusion as an invitation—and toward empowerment as shared action, voice, and power.

## Data Availability

The raw data supporting the conclusions of this article will be made available by the authors, without undue reservation.
